# Gender Gap in Parental Leave Among Physicians in Japan

**DOI:** 10.1089/whr.2023.0126

**Published:** 2024-05-03

**Authors:** Kayo Fukami

**Affiliations:** National Institute of Technology, Toba College, Toba, Japan.

**Keywords:** gender gap, medical sociology, parental leave, career, female physician

## Abstract

**Objective:**

To investigate the gender gap in parental leave uptake among physicians and explore the burden of childcare on female physicians compared with their male counterparts.

**Methods:**

The focus was on the rate for taking childcare leave as an indicator of the gender gap in the burden of childcare. Data from the Japanese Ministry of Health, Labor and Welfare’s national database were analyzed to investigate the population ratio of physicians who took parental leave. The study included male and female physicians from different years and prefectures.

**Results:**

Gender disparity in parental leave uptake among physicians was observed. On average, male physicians take parental leave at a rate of 0.05%, while female physicians have a much higher rate of 4.5%. Around 1,400 to 1,700 female physicians took parental leave annually, compared with only 20–70 male physicians. This highlights the disproportionate burden of childcare on female physicians.

**Conclusion:**

The study demonstrates a considerable childcare burden on female physicians due to the rarity of male physicians taking parental leave. The findings underscore the urgency of addressing the gender gap in parental leave uptake among physicians and promoting gender equality in childcare responsibilities. Future research and policy initiatives should focus on achieving a more equitable distribution of parental leave to alleviate the burden on female healthcare professionals and improve work-life balance in the medical profession.

## Introduction

The Organization for Economic Cooperation and Development (OECD) has reported that nearly 50% of physicians in various countries are female.^1^ However, despite this significant progress, maternity and parental leave systems remain inadequate. In the United States, the 1993 Family Medical Leave Act (FMLA) requires employers (with 50 or more employees) to provide up to 12 weeks of unpaid leave, including parental leave, to eligible employees (those who have worked at least 1,250 hours in the previous 12 months). However, the leave is unpaid, and individual offices have the authority to determine the provisions for maternity leave.^2^ Many workplaces and residency programs lack maternity leave or paid parental leave.^3,4^ According to a descriptive study conducted by the Council of Academic Medicine Educational Research Alliance in 2017, maternity leave policies in U.S. family medicine residency programs differ in their duration of paid maternity and parental leaves, ranging from 0 to 12 weeks. Almost 20% of surveyed programs did not offer maternity leave, and nearly 40% did not provide paid maternity leave to both faculty and residents. Only two programs provided more than 12 weeks of paid maternity leave to residents and faculty.^3^ A survey conducted in 2007 found that only 42% of female urologists had a formal maternity leave policy, with 70% taking eight weeks of leave or less.^5^ Although Canada has an exceptional social security system, physicians seeking leave face the challenge of negotiating directly with their supervisors, workplaces, and mentors due to the lack of consistent guidelines.^6^

Social security for residents’ maternity and childcare is limited. Maternity and paternal leave policies generally cover employed persons, yet 67% of the respondents became parents during residency.^7^ In the U.S., residents face challenges when planning for pregnancy during their residency, leading to conflicts.^3,8–11^ They face institutional shortcomings and negative influences early in their careers, resulting in an average parental leave duration of nearly three weeks less than that taken by physicians.^12^ Pregnancy during residency is associated with complications such as gestational hypertension, placental abruption, preterm delivery, and an increased risk of intrauterine growth retardation.^13,14^ Female residents were approximately three times more likely than the partners of male residents to selectively terminate a pregnancy.^15^ Residents face challenges with time, and financial and psychological pressures.

Gender stereotypes and the workplace culture of the medical community hinder physicians’ engagement in childbirth and childcare,^16,17^ despite adequate systems in place. Multiple reviews on physician pregnancy persistently highlight the discrimination, stigma, and inconsistent implementation of leave policies.^18–20^ Even in the United Kingdom, where the social security system is relatively well developed, physicians trying to raise children feel “out of place for unidentifiable reasons” or explicit sexism.^17^ Similarly, in Canada, the medical culture remains inflexible and unwelcoming toward pregnancy and parenting.^9^ Stigma surrounding pregnancy and childbirth among physicians has been reported in Japan.^21^ A survey by the Japan Surgical Society found that 62% of female surgeons received negative comments about pregnancy.^22^

Male physicians face distinct challenges when it comes to childcare involvement due to gender stereotypes. In many countries (*n* = 573), male parental leave is rare (*n* = 11, 2.6%),^7^ and male physicians feel guilty about taking leave.^23^ In Japan, some work–life balance policies target only female doctors, reinforcing traditional gender roles, with men working long hours and women assuming domestic work.^24^ Therefore, it can be expected that the taking of childcare leave is also unevenly biased toward women.

The extent of the gender gap in parental leave uptake in Japan is unclear. Institutionally, the country has a well-developed parental leave system for men that offers more than six months of leave, the highest among countries in UNICEF’s family-friendly leave ranking.^25^ Maternity leave is regulated by Article 65 of the Labor Standards Law, while parental leave for both genders is defined in the Child Care and Family Care Leave Law. Despite equal guarantees, a significant gender divide persists, with 81.6% of women taking parental leave compared with only 12.65% of men.^26^

In the medical community, childcare responsibilities are disproportionately shouldered by women. Among 427 surveyed female neurosurgeons in Japan, 17% quit their full-time positions, with 52% citing challenges in balancing their career and motherhood roles.^27^ Moreover, male physicians tend to work full-time regardless of their spouse’s occupation, while female physicians have a lower rate of full-time work if their spouse is also a physician.^28^ In physician couples, the female partner bears the burden of career restraint.

Guaranteeing access to parental leave is important for assuring the right to have children and for work–life balance, and it needs to be expanded. However, if the users of the system are predominantly women, this implies a reproduction of the classic gendered division of labor. Therefore, in this study, the actual situation of the childcare burden was analyzed by considering the gender gap in the rate of taking childcare leave as an indicator of the gap in childcare responsibilities.

The existing literature is largely limited to surveys of single institutions or facilities and is primarily centered around female physicians. To the best of my knowledge, no study has been conducted to identify and explore the gender gap associated with the national parental leave-taking rate. To investigate the gender disparity in physicians taking parental leave, data were obtained from the 2016 *Statistics of Physicians, Dentists, and Pharmacists* as well as the 2018 and 2020 *Survey of Physicians Dentists and Pharmacists*. These sources constitute the most extensive database of physicians in Japan.

## Materials and Methods

### Data collection and study population

Japan’s Ministry of Health, Labor and Welfare (MHLW)’s *Survey of Physicians, Dentists and Pharmacists* and *Statistics of Physicians, Dentists and Pharmacists* are the largest databases on physicians in the country. The databases are updated every alternate year. The year-wise updated data of the number of physicians were 319,480 in 2016, 327,210 in 2018, and 339,623 in 2020.

A new item on leave (maternity, paternity, and nursing care leave) was added in the 2016 survey. For this analysis, I obtained all available data for three years: the 2016 edition of the *Survey of Physicians, Dentists and Pharmacists* and the 2018 and 2020 editions of the *Statistics of Physicians, Dentists and Pharmacists*. This database, *Survey of Physicians, Dentists and Pharmacists*, was renamed *Statistics of Physicians, Dentists and Pharmacists* in 2018. The two databases are almost the same. The renaming occurred due to an update in the Statistic law. This study examined the gender gap in childcare among physicians, with a focus on parental leave available to both men and women. The analysis targeted the age group of 25–39 years, representing more than 86% of those women taking parental leave. The basic data of the analyzed subjects are shown in Table 1. This study focuses more on the gender gap that accumulates from the beginning of one’s career, and the number of people taking parental leave is extremely small for those older than 50 years. This population was excluded because those data are not suitable for a statistical analysis.

**Table 1.** Basic Data of the Analyzed Participants (25–39 Years Old)

	2016		2018		2020		2016		2018		2020		2016		2018		2020	
	Total	Parental leave (pop.)	Total	Parental leave (pop.)	Total	Parental leave (pop.)	Total	Parental leave (pop.)	Total	Parental leave (pop.)	Total	Parental leave (pop.)	Total	Parental leave (pop.)	Total	Parental leave (pop.)	Total	Parental leave (pop.)
Nationwide							16 Toyama						32 Shimane					
Total	94,031	1,311	95,404	1,539	99,494	1,559	662	9	703	2	755	13	490	17	527	13	559	11
Male	63,573	10	64,230	37	66,789	45	461	0	497	0	522	0	320	0	340	0	363	0
Female	30,458	1,301	31,174	1,502	32,705	1,514	201	9	206	2	233	13	170	17	187	13	196	11
1 Hokkaido							17 Ishikawa						33 Okayama					
Total	3,401	20	3,378	31	3,490	42	1,040	14	1,026	47	1,051	21	1,822	22	1,848	25	1,929	21
Male	2,543	0	2,463	0	2,547	7	733	0	723	24	746	0	1,252	0	1,263	1	1,314	1
Female	858	20	915	31	943	35	307	14	303	23	305	21	570	22	585	24	615	20
Total	666	9	668	5	711	8	566	10	597	7	600	8	1,857	23	1,836	28	1,929	27
Male	458	0	460	0	469	0	388	0	404	0	406	0	1,250	0	1,259	0	1,295	0
Female	208	9	208	5	242	8	178	10	193	7	194	8	607	23	577	28	634	27
3 Iwate							19 Yamanashi						35 Yamaguchi					
Total	672	5	668	15	690	5	533	3	533	6	605	2	773	5	769	8	781	12
Male	496	0	473	0	506	0	384	0	376	0	412	0	544	0	538	0	547	0
Female	176	5	195	15	184	5	149	3	157	6	193	2	229	5	231	8	234	12
4 Miyagi							20 Nagano						36 Tokushima					
Total	1,631	23	1,649	34	1,736	26	1,245	17	1,255	18	1,342	15	599	11	618	15	616	20
Male	1,164	0	1,185	0	1,218	0	904	1	918	0	969	0	383	1	375	0	387	1
Female	467	23	464	34	518	26	341	16	337	18	373	15	216	10	243	15	229	19
5 Akita							21 Gifu						37 Kagawa					
Total	654	10	686	12	691	5	1,191	18	1,209	18	1,262	15	702	16	706	13	706	14
Male	457	0	463	1	458	0	880	0	889	0	921	0	461	0	482	0	479	0
Female	197	10	223	11	233	5	311	18	320	18	341	15	241	16	224	13	227	14
6 Yamagata							22 Shizuoka						38 Ehime					
Total	692	8	701	13	712	13	2,252	24	2,318	27	2,484	22	901	13	910	8	935	19
Male	518	0	517	0	539	0	1,668	0	1,736	0	1,828	0	611	1	609	0	629	0
Female	174	8	184	13	173	13	584	24	582	27	656	22	290	12	301	8	306	19
7 Fukushima							23 Aichi						39 Kochi					
Total	906	10	952	20	975	12	5,414	96	5,508	98	5,702	87	556	12	576	9	591	10
Male	682	0	696	0	696	0	3,690	0	3,754	1	3,885	2	349	0	370	0	379	0
Female	224	10	256	20	279	12	1,724	96	1,754	97	1,817	85	207	12	206	9	212	10
8 Ibaraki							24 Mie						40 Fukuoka					
Total	1,682	14	1,761	29	1,757	15	1,001	18	1,032	26	1,091	27	5,058	66	4,988	84	5,078	98
Male	1,156	1	1,209	0	1,177	1	736	0	749	1	772	0	3,466	1	3,393	1	3,502	2
Female	526	13	552	29	580	14	265	18	283	25	319	27	1,592	65	1,595	83	1,576	96
9 Tochigi							25 Shiga						41 Saga					
Total	1,419	19	1,461	26	1,563	30	992	18	1,035	16	1,043	16	702	8	666	16	687	18
Male	970	0	985	1	1,072	0	667	0	717	0	694	0	451	0	435	0	439	0
Female	449	19	476	25	491	30	325	18	318	16	349	16	251	8	231	16	248	18
10 Gunma							26 Kyoto						42 Nagasaki					
Total	1,172	19	1,117	18	1,155	28	2,940	28	2,881	36	2,901	35	997	11	1,042	20	1,086	13
Male	794	0	768	0	811	0	2,047	0	1,976	0	2,009	2	641	0	683	0	715	0
Female	378	19	349	18	344	28	893	28	905	36	892	33	356	11	359	20	371	13
11 Saitama							27 Osaka						43 Kumamoto					
Total	3,237	47	3,533	53	3,748	50	7,524	111	7,613	95	7,815	127	1,233	23	1,267	23	1,256	27
Male	2,246	0	2,405	1	2,512	0	4,976	0	4,951	0	5,121	8	848	0	863	0	848	1
Female	991	47	1,128	52	1,236	50	2,548	111	2,662	95	2,694	119	385	23	404	23	408	26
12 Chiba							28 Hyogo						44 Oita					
Total	3,822	49	3,887	45	4,224	46	3,922	61	4,022	70	4,338	72	742	11	742	23	776	12
Male	2,607	0	2,691	0	2,918	2	2,630	0	2,706	0	2,860	3	490	0	493	0	540	0
Female	1,215	49	1,196	45	1,306	44	1,292	61	1,316	70	1,478	69	252	11	249	23	236	12
13 Tokyo							29 Nara						45 Miyazaki					
Total	15,819	246	16,117	310	16,786	311	944	17	1,038	16	1,107	18	591	9	599	11	674	13
Male	9,806	4	9,966	4	10,373	11	659	0	734	0	791	0	383	0	395	0	454	0
Female	6,013	242	6,151	306	6,413	300	285	17	304	16	316	18	208	9	204	11	220	13
14 Kanagawa							30 Wakayama						46 Kagoshima					
Total	6,548	89	6,556	91	6,850	98	793	13	813	10	862	15	961	9	994	14	1,046	12
Male	4,281	1	4,245	1	4,396	1	547	0	565	0	601	0	681	0	706	0	727	0
Female	2,267	88	2,311	90	2,454	97	246	13	248	10	261	15	280	9	288	14	319	12
15 Niigata							31 Tottori						47 Okinawa					
Total	1,112	12	1,138	13	1,152	12	456	2	447	6	482	7	1,139	16	1,014	16	1,165	31
Male	804	0	813	0	824	0	325	0	315	0	339	0	766	0	677	1	779	3
Female	308	12	325	13	328	12	131	2	132	6	143	7	373	16	337	15	386	28

This study was conducted with published data; therefore, it was not necessary to obtain informed consent. The Ethics Committee of the National Institute of Technology, Toba College, confirmed that ethical approval was not required for this study.

### Analysis

Taking the rate of parental leave as an indicator of gender disparity in parental responsibility, parental leave rates were estimated by prefecture and gender for the years 2016–2020, along with their corresponding 99% confidence intervals (CI). A population ratio test was conducted using the null hypothesis that the average rate of parental leave is equal for men and women, and used a *z*-test to assess whether parental leave behavior was the same for men and women in each prefecture. The significance level of α = 0.01 was applied, and the average rate of women taking parental leave was considered to be significantly higher when the test statistic was smaller than the rejection region of −2.33. Data were analyzed in February 2023 using Microsoft Excel for Windows (statistical software version 14.0.7268.5000, Microsoft Corp).

As it is assumed that there may be differences in parental leave-taking behaviors depending on the prefecture of residence, data were analyzed by prefecture. The decision to take parental leave depends on the existence of available childcare centers. In Japan, birth rates tend to be higher in the western half of the country, and the availability of childcare centers varies accordingly from prefecture to prefecture. Living in prefectures with well-equipped childcare centers makes it easier to balance work and childcare, so it can be assumed that the gender gap in the parental leave rate in such areas is smaller. To confirm this assumption, this article analyzes the situation by prefecture.

## Results

### Demographic data

Table 1 presents the demographic data of the physicians and the frequency of taking parental leave across prefectures in 2016, 2018, and 2020.

### Statistical analysis

Table 2 summarizes the results of nationwide population ratio tests for people taking parental leave in 2016, 2018, and 2020, along with the number of physicians by gender. It also shows the three-year average rate of people taking parental leave and 99% CI. The test was based on the null hypothesis that assumed equal rates of parental leave for a men and women, with a significance level of 1%. Women had a significantly higher percentage of taking leave nationally. This trend was observed similarly in all prefectures. The results by prefecture are presented in the Supplementary Table S1. Table 3 presents the total number of female and male physicians who took parental leave, categorized by age group, in the years 2016, 2018, and 2020.

**Table 2.** Nationwide Results of the Population Ratio Tests of Taking Parental Leave

	Average cases of parental leave	Confidence interval	z-value
Nationwide			
Male	0.05	(CI: 0.00-0.10)	−48.67
Female	4.57	(CI: 0.44-8.71)	

**Table 3.** Number of Female and Male Physicians Who Took Parental Leave, Categorized by Age Group and Year

Female				Male			
Age	2016	2018	2020	Age	2016	2018	2020
<24	—	—	—	<24	—	—	1
25–29	116	175	153	25–29	1	17	7
30–34	628	682	712	30–34	5	7	20
35–39	557	645	649	35–39	4	13	18
40–44	156	210	218	40–44	6	4	10
45–49	8	17	15	45–49	1	7	3
50–54	2	2	—	50–54	1	3	5
55–59	—	—	—	55–59	—	5	4
60–64	1	—	—	60–64	1	3	4

The average rate of taking parental leave was 0.05% for men and 4.57% for women (Table 2). From 2018 to 2020, the number of men and women taking parental leave slightly increased nationwide for most age groups, while that of younger physicians (25-29 years) decreased (Table 3). COVID-19 may be a reason for this. Younger physicians with preschool-age children have been more likely to leave work due to day care closures and child-related issues. This could create more opportunities for taking other types of leave than parental leave. Unlike women, men took very little parental leave in all prefectures in all years. While the area of residence influences maternity and childcare, the tendency for men not to take parental leave is the same in all prefectures (Supplementary Table S1).

## Discussion

Using the national database, this study revealed a significant gender gap in the average rate of physicians taking parental leave: 0.05% for male and 4.57% for female (Table 2). Nationwide, approximately 1,400–1,700 female physicians take parental leave annually compared with only 20–70 of their male counterparts, highlighting the imbalance in their childcare burden (Table 3). Previous research confirms that in many countries, male physicians rarely take parental leave^7^ and feel guilty when they do,^23^ aligning with the results of this study. This study found that in all 47 prefectures, the percentage of female physicians taking parental leave was significantly higher (Supplementary Table S1). The gender gap in the childcare burden between men and women was not due to inadequate social systems but rather to the division of gender roles.

For female physicians in Japan, having children has a major impact on their careers. A survey of 427 female neurosurgeons in Japan found that 17% quit their full-time positions, with half of that 17% citing challenges of balancing work with motherhood as the primary reason.^27^ The work rate of female physicians is lower when their husbands are physicians than in other professions, indicating that in physician couples, the female physician shoulders the entire burden of childcare.^28^ This does not mean that the male parental leave system is lacking. In Japan, both men and women are entitled to one year of parental leave, which is longer than in many countries.^25^ During this period, up to 67% of the pre-leave wage is provided as wage security.^29^ However, a survey of the general workforce showed that women take 81.6% of that parental leave, while men take 12.65%, indicating that the overall trend in Japan is for women to bear a disproportionate burden of childcare.^26^

The gender gap in the childcare burden of physicians is often reported for medical students up to around their 40s. According to these reports, careers begin to suggest significant disparities already at anearly stage, even if they appear to have started their careers at the same time. In the Netherlands, for example, it has been reported that male medical students tend to prefer a “less ambitious” future partner.^30^ It can be argued that they envisage a future in which their career takes priority, when they have a family. In many countries, the residency years coincide with peak child-rearing years, and female residents who have given birth tend to carry most of the child-rearing responsibilities.^31^ Female residents tend to intentionally delay pregnancy to avoid potential threats to their careers, while men at the same career level try to have children.^11^ Due to gender inequalities accumulated since the beginning of the career path, male physicians are more likely to be married and have children. The impact of family life on careers differs significantly between men and women.^32^

Although not subject to statistical analysis in this study, simple aggregate results reveal that 15.7–30.5% of male physicians who opted for parental leave did so at the age of 45 or older (Table 3). Since 69%–84% of male physicians who took parental leave were 44 years or younger, it can be said that those 44 years or younger comprise the volume zone for those taking parental leave. For female physicians as well, 99% of those taking parental leave are 44 years or younger. However, in 2020, for example, parental leave was taken by three male physicians aged 45–49, five men aged 50–54, four men aged 55–59, and four men aged 60–64, indicating that some male physicians take parental leave at the age of 45 or older. In contrast, 15 female doctors aged 45–49 took childcare leave, and none aged 50 or older took such leave. Male physicians, unlike female physicians, whose pregnancies often coincide with the early stages of their careers, have more flexibility in scheduling parental leave, and for men, having a child may be a life event that is not restricted by their own age. This suggests a gender disparity in terms of career flexibility.

### Limitations

This study included all physicians rather than only physicians with children. Male physicians tend to have higher fertility rates compared with their female counterparts, which means that the gender gap in parental leave rates may have been underestimated.

## Conclusions

To assess gender disparities in physicians’ parental leave, a mother’s ratio test was conducted using the parental leave-taking rate as an indicator of the childcare burden gap. The results revealed that female physicians were more likely to take parental leave across different prefectures and years. The average rate of male physicians taking parental leave is 0.05%, while for female physicians, it is 4.57%, indicating a significant gender gap. In addition, regardless of their location, the study found that the percentage of female physicians taking parental leave was significantly higher in all 47 prefectures. Around 1,400–1,700 female physicians take parental leave annually in Japan, while only about 20–70 male physicians do so. The burden of childcare is heavily skewed toward female physicians despite the relatively well-developed childcare leave system. This gender disparity is believed to stem from gender stereotypes. In addition, male physicians were found to take parental leave even after the age of 45. This suggests that they have an advantage over women in that they can flexibly decide at what stage in their life to have children.

## Supplementary Material

Supplementary Table S1

## Data Availability

All data and materials supporting this article’s conclusions are available on the internet. Examined data were obtained from the official website of Japan’s MHLW.

## Abbreviations Used

CI: confidence interval
FMLA: Family Medical Leave Act
MHLW: Ministry of Health, Labour and Welfare
OECD: Organisation for Economic Cooperation and Development
