# Supplementary material for: Gender Gap in Parental Leave Among Physicians in Japan
Source: Womens Health Rep (New Rochelle). 2024 May 3;5(1):385–92. doi: 10.1089/whr.2023.0126 (PMC11257144; doi:10.1089/whr.2023.0126)
Supplement: Supplementary Table S1 [file whr.2023.0126_supp_tables1.docx]

**Supplementary table**. Results of the population ratio tests of taking parental leave by **prefecture** and gender

|  |  | Ave. cases of parental leave | Confidence interval | | | | | *z-value* |  |  | Ave. cases of parental leave | Confidence interval | | | | | *z-value* |  |  | Ave. cases of parental leave | Confidence interval | | | | | *z-value* |
| --- | --- | --- | --- | --- | --- | --- | --- | --- | --- | --- | --- | --- | --- | --- | --- | --- | --- | --- | --- | --- | --- | --- | --- | --- | --- | --- |
|  | Nationwide |  |  |  |  |  |  |  | 16 | Toyama |  |  |  |  |  |  |  | 32 | Shimane |  |  |  |  |  |  |  |
|  | Male | 0.05 | (CI: | 0.00 | - | 0.10 | ) | -48.67 |  | Male | 0.00 | (CI: | 0.00 | - | 0.00 | ) | -4.58 |  | Male | 0.00 | (CI: | 0.00 | - | 0.00 | ) | -10.03 |
|  | Female | 4.57 | (CI: | 0.44 | - | 8.71 | ) |  |  | Female | 3.68 | (CI: | -0.48 | - | 7.83 | ) |  |  | Female | 7.52 | (CI: | 0.35 | - | 14.70 | ) |  |
| 1 | Hokkaido |  |  |  |  |  |  |  | 17 | Ishikawa |  |  |  |  |  |  |  | 33 | Okayama |  |  |  |  |  |  |  |
|  | Male | 0.09 | (CI: | -0.09 | - | 0.28 | ) | -12.39 |  | Male | 1.11 | (CI: | -1.12 | - | 3.34 | ) | -6.32 |  | Male | 0.05 | (CI: | -0.01 | - | 0.12 | ) | -25.11 |
|  | Female | 3.14 | (CI: | 0.21 | - | 6.08 | ) |  |  | Female | 6.35 | (CI: | 0.39 | - | 12.30 | ) |  |  | Female | 3.74 | (CI: | 0.34 | - | 7.14 | ) |  |
| 2 | Aomori |  |  |  |  |  |  |  | 18 | Fukui |  |  |  |  |  |  |  | 34 | Hiroshima |  |  |  |  |  |  |  |
|  | Male | 0.00 | (CI: | 0.00 | - | 0.00 | ) | -10.43 |  | Male | 0.00 | (CI: | 0.00 | - | 0.00 | ) | -12.90 |  | Male | 0.00 | (CI: | 0.00 | - | 0.00 | ) | -24.20 |
|  | Female | 3.35 | (CI: | 0.17 | - | 6.52 | ) |  |  | Female | 4.46 | (CI: | 0.30 | - | 8.62 | ) |  |  | Female | 4.30 | (CI: | 0.38 | - | 8.22 | ) |  |
| 3 | Iwate |  |  |  |  |  |  |  | 19 | Yamanashi |  |  |  |  |  |  |  | 35 | Yamaguchi |  |  |  |  |  |  |  |
|  | Male | 0.00 | (CI: | 0.00 | - | 0.00 | ) | -4.67 |  | Male | 0.00 | (CI: | 0.00 | - | 0.00 | ) | -4.86 |  | Male | 0.00 | (CI: | 0.00 | - | 0.00 | ) | -7.30 |
|  | Female | 4.42 | (CI: | -0.54 | - | 9.37 | ) |  |  | Female | 2.29 | (CI: | -0.25 | - | 4.83 | ) |  |  | Female | 3.59 | (CI: | 0.01 | - | 7.18 | ) |  |
| 4 | Miyagi |  |  |  |  |  |  |  | 20 | Nagano |  |  |  |  |  |  |  | 36 | Tokushima |  |  |  |  |  |  |  |
|  | Male | 0.00 | (CI: | 0.00 | - | 0.00 | ) | -12.69 |  | Male | 0.04 | (CI: | -0.04 | - | 0.11 | ) | -21.03 |  | Male | 0.17 | (CI: | -0.05 | - | 0.39 | ) | -10.06 |
|  | Female | 5.76 | (CI: | 0.38 | - | 11.14 | ) |  |  | Female | 4.68 | (CI: | 0.41 | - | 8.96 | ) |  |  | Female | 6.37 | (CI: | 0.31 | - | 12.42 | ) |  |
| 5 | Akita |  |  |  |  |  |  |  | 21 | Gifu |  |  |  |  |  |  |  | 37 | Kagawa |  |  |  |  |  |  |  |
|  | Male | 0.07 | (CI: | -0.07 | - | 0.22 | ) | -7.21 |  | Male | 0.00 | (CI: | 0.00 | - | 0.00 | ) | -20.82 |  | Male | 0.00 | (CI: | 0.00 | - | 0.00 | ) | -44.43 |
|  | Female | 4.05 | (CI: | 0.01 | - | 8.09 | ) |  |  | Female | 5.27 | (CI: | 0.45 | - | 10.09 | ) |  |  | Female | 6.20 | (CI: | 0.59 | - | 11.81 | ) |  |
| 6 | Yamagata |  |  |  |  |  |  |  | 22 | Shizuoka |  |  |  |  |  |  |  | 38 | Ehime |  |  |  |  |  |  |  |
|  | Male | 0.00 | (CI: | 0.00 | - | 0.00 | ) | -12.21 |  | Male | 0.00 | (CI: | 0.00 | - | 0.00 | ) | -18.73 |  | Male | 0.05 | (CI: | -0.06 | - | 0.16 | ) | -7.19 |
|  | Female | 6.39 | (CI: | 0.40 | - | 12.38 | ) |  |  | Female | 4.03 | (CI: | 0.34 | - | 7.73 | ) |  |  | Female | 4.33 | (CI: | 0.01 | - | 8.66 | ) |  |
| 7 | Fukushima |  |  |  |  |  |  |  | 23 | Aichi |  |  |  |  |  |  |  | 39 | Kochi |  |  |  |  |  |  |  |
|  | Male | 0.00 | (CI: | 0.00 | - | 0.00 | ) | -8.36 |  | Male | 0.03 | (CI: | -0.01 | - | 0.06 | ) | -31.14 |  | Male | 0.00 | (CI: | 0.00 | - | 0.00 | ) | -19.99 |
|  | Female | 5.53 | (CI: | 0.13 | - | 10.92 | ) |  |  | Female | 5.26 | (CI: | 0.49 | - | 10.03 | ) |  |  | Female | 4.96 | (CI: | 0.42 | - | 9.50 | ) |  |
| 8 | Ibaraki |  |  |  |  |  |  |  | 24 | Mie |  |  |  |  |  |  |  | 40 | Fukuoka |  |  |  |  |  |  |  |
|  | Male | 0.06 | (CI: | -0.02 | - | 0.13 | ) | -6.14 |  | Male | 0.04 | (CI: | -0.05 | - | 0.13 | ) | -21.97 |  | Male | 0.04 | (CI: | 0.00 | - | 0.08 | ) | -15.16 |
|  | Female | 3.38 | (CI: | -0.10 | - | 6.86 | ) |  |  | Female | 8.03 | (CI: | 0.70 | - | 15.36 | ) |  |  | Female | 5.13 | (CI: | 0.39 | - | 9.86 | ) |  |
| 9 | Tochigi |  |  |  |  |  |  |  | 25 | Shiga |  |  |  |  |  |  |  | 41 | Saga |  |  |  |  |  |  |  |
|  | Male | 0.03 | (CI: | -0.03 | - | 0.10 | ) | -16.44 |  | Male | 0.00 | (CI: | 0.00 | - | 0.00 | ) | -31.75 |  | Male | 0.00 | (CI: | 0.00 | - | 0.00 | ) | -7.68 |
|  | Female | 5.20 | (CI: | 0.41 | - | 9.99 | ) |  |  | Female | 5.05 | (CI: | 0.47 | - | 9.63 | ) |  |  | Female | 5.79 | (CI: | 0.06 | - | 11.52 | ) |  |
| 10 | Gunma |  |  |  |  |  |  |  | 26 | Kyoto |  |  |  |  |  |  |  | 42 | Nagasaki |  |  |  |  |  |  |  |
|  | Male | 0.00 | (CI: | 0.00 | - | 0.00 | ) | -10.41 |  | Male | 0.03 | (CI: | -0.03 | - | 0.10 | ) | -24.74 |  | Male | 0.00 | (CI: | 0.00 | - | 0.00 | ) | -9.15 |
|  | Female | 6.11 | (CI: | 0.30 | - | 11.91 | ) |  |  | Female | 3.60 | (CI: | 0.32 | - | 6.88 | ) |  |  | Female | 4.05 | (CI: | 0.15 | - | 7.96 | ) |  |
| 11 | Saitama |  |  |  |  |  |  |  | 27 | Osaka |  |  |  |  |  |  |  | 43 | Kumamoto |  |  |  |  |  |  |  |
|  | Male | 0.01 | (CI: | -0.01 | - | 0.04 | ) | -35.99 |  | Male | 0.05 | (CI: | -0.05 | - | 0.16 | ) | -25.29 |  | Male | 0.04 | (CI: | -0.04 | - | 0.12 | ) | -51.48 |
|  | Female | 4.47 | (CI: | 0.42 | - | 8.51 | ) |  |  | Female | 4.11 | (CI: | 0.37 | - | 7.86 | ) |  |  | Female | 6.01 | (CI: | 0.58 | - | 11.45 | ) |  |
| 12 | Chiba |  |  |  |  |  |  |  | 28 | Hyogo |  |  |  |  |  |  |  | 44 | Oita |  |  |  |  |  |  |  |
|  | Male | 0.02 | (CI: | -0.02 | - | 0.07 | ) | -33.01 |  | Male | 0.03 | (CI: | -0.04 | - | 0.11 | ) | -39.86 |  | Male | 0.00 | (CI: | 0.00 | - | 0.00 | ) | -7.11 |
|  | Female | 3.72 | (CI: | 0.35 | - | 7.09 | ) |  |  | Female | 4.90 | (CI: | 0.47 | - | 9.34 | ) |  |  | Female | 6.23 | (CI: | -0.02 | - | 12.48 | ) |  |
| 13 | Tokyo |  |  |  |  |  |  |  | 29 | Nara |  |  |  |  |  |  |  | 45 | Miyazaki |  |  |  |  |  |  |  |
|  | Male | 0.06 | (CI: | -0.01 | - | 0.13 | ) | -27.67 |  | Male | 0.00 | (CI: | 0.00 | - | 0.00 | ) | -47.80 |  | Male | 0.00 | (CI: | 0.00 | - | 0.00 | ) | -19.37 |
|  | Female | 4.56 | (CI: | 0.42 | - | 8.70 | ) |  |  | Female | 5.64 | (CI: | 0.54 | - | 10.74 | ) |  |  | Female | 5.21 | (CI: | 0.44 | - | 9.98 | ) |  |
| 14 | Kanagawa |  |  |  |  |  |  |  | 30 | Wakayama |  |  |  |  |  |  |  | 46 | Kagoshima |  |  |  |  |  |  |  |
|  | Male | 0.02 | (CI: | 0.00 | - | 0.04 | ) | -308.06 |  | Male | 0.00 | (CI: | 0.00 | - | 0.00 | ) | -16.98 |  | Male | 0.00 | (CI: | 0.00 | - | 0.00 | ) | -14.11 |
|  | Female | 3.91 | (CI: | 0.38 | - | 7.43 | ) |  |  | Female | 5.02 | (CI: | 0.40 | - | 9.64 | ) |  |  | Female | 3.95 | (CI: | 0.28 | - | 7.61 | ) |  |
| 15 | Niigata |  |  |  |  |  |  |  | 31 | Tottori |  |  |  |  |  |  |  | 47 | Okinawa |  |  |  |  |  |  |  |
|  | Male | 0.00 | (CI: | 0.00 | - | 0.00 | ) | -66.01 |  | Male | 0.00 | (CI: | 0.00 | - | 0.00 | ) | -5.92 |  | Male | 0.18 | (CI: | -0.08 | - | 0.44 | ) | -9.21 |
|  | Female | 3.85 | (CI: | 0.37 | - | 7.33 | ) |  |  | Female | 3.66 | (CI: | -0.16 | - | 7.47 | ) |  |  | Female | 5.33 | (CI: | 0.22 | - | 10.44 | ) |  |
